# TRIMming down to TRIM37: Relevance to Inflammation, Cardiovascular Disorders, and Cancer in MULIBREY Nanism

**DOI:** 10.3390/ijms20010067

**Published:** 2018-12-24

**Authors:** Benjamin Brigant, Valérie Metzinger-Le Meuth, Jacques Rochette, Laurent Metzinger

**Affiliations:** 1 HEMATIM, EA4666, CURS, CHU Amiens Sud, Avenue René Laënnec, Salouel, F-80054 Amiens, France; benjamin.brigant@gmail.com (B.B.); Laurent.metzinger@u-picardie.fr (L.M.); 2 INSERM U1148, Laboratory for Vascular Translational Science (LVTS), UFR SMBH, Université Paris 13-Sorbonne Paris Cité, 93017 Bobigny CEDEX, France; valerie.metzinger@univ-paris13.fr

**Keywords:** mulibrey, TRIM37, pericarditis, NF-κB, ubiquitin

## Abstract

TRIpartite motif (TRIM) proteins are part of the largest subfamilies of E3 ligases that mediate the transfer of ubiquitin to substrate target proteins. In this review, we focus on TRIM37 in the normal cell and in pathological conditions, with an emphasis on the MULIBREY (MUscle-LIver-BRain-EYe) genetic disorder caused by *TRIM37* mutations. TRIM37 is characterized by the presence of a RING domain, B-box motifs, and a coiled-coil region, and its C-terminal part includes the MATH domain specific to TRIM37. MULIBREY nanism is a rare autosomal recessive caused by *TRIM37* mutations and characterized by severe pre- and postnatal growth failure. Constrictive pericarditis is the most serious anomaly of the disease and is present in about 20% of patients. The patients have a deregulation of glucose and lipid metabolism, including type 2 diabetes, fatty liver, and hypertension. Puzzlingly, MULIBREY patients, deficient for TRIM37, are plagued with numerous tumors. Among non-MULIBREY patients affected by cancer, a wide variety of cancers are associated with an overexpression of TRIM37. This suggests that normal cells need an optimal equilibrium in TRIM37 expression. Finding a way to keep that balance could lead to potential innovative drugs for MULIBREY nanism, including heart condition and carcinogenesis treatment.

## 1. Introduction

In 2001, Reymond et al. used functional genomic techniques to show that the TRIpartite motif (TRIM), with the presence of a RING domain, B-box motifs, and a coiled-coil region, defines a family of 37 proteins with common cellular functions and compartmentalization. These proteins were regrouped in the TRIM superfamily (TRIpartite motif) [1]. Other similar proteins were added over time to the family, and it emerged that *TRIM* genes arose from a common ancestor and can be found in most eukaryotes. The tripartite motif is restricted to metazoans, and there is a wide variation between species in the number of TRIM proteins, which range from 78 in humans to much less in worms (20) and flies (10). This suggests an extensive adaption over time of the TRIM family in vertebrates and invertebrates, with an important maintenance throughout evolution together with an expansion of novel *TRIM* genes engaged in a wider diversity of functions.

**Figure 1:**
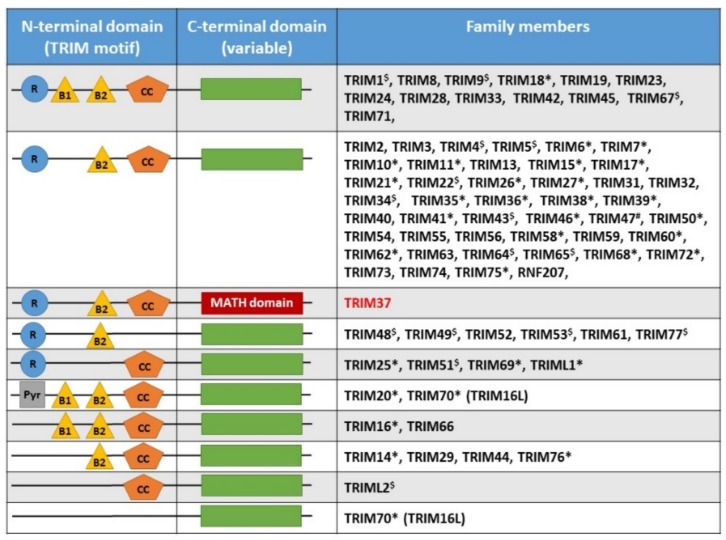
Comparison of the organization and classification of TRIM (TRIpartite motif) proteins. MATH domain is specific for TRIM37. R: RING-finger domain, B1: B-box domain 1, B2: B-box domain 2, CC: coiled-coil domain, Pyr: pyrin, domain MATH (meprin and TRAF-homology domain). * indicates PRY/SPRY domain, # indicates PRY domain, and $ indicates SPRY domain. Adapted from Reference [2]. Group 1 is represented by # or absence of symbol. Group 2 is represented by $ and *.

As previously stated, TRIM proteins are characterized by different subdomains, including a N-terminal domain containing the following: (i) a RING domain, a unique linear series of conserved cysteine and histidine residues of a zinc finger type that binds a pair of zinc atoms and is involved in mediating protein–protein interactions; (ii) one or two B-box motifs built of small peptide sequences containing finger-like protrusions involved in target protein recognition; and (iii) a coiled-coil region that mediates TRIM homo- or oligodimerization. (Figure 1) [2]. The TRIM motif is followed by variable C-terminal domains, which constitute a specific functional unit and are often used to classify TRIM family members into subgroups when the RING motif is the central catalytic domain. It should be noted that not all TRIM proteins in humans have a ring-finger domain. The C-terminal portion displays nucleic-acid-binding properties and specific enzymatic activities. Depending on the organism and the protein, the C-terminal domain contains two regions: either in combination or individually. One is made of an approximately 61 amino-acids-long sequence (the PRY domain), and the other one contains a sequence that is approximately 160 amino acids long (the SPRY domain). The PRY–SPRY domains are found in over 500 different proteins, which are involved in proliferation, innate immune response, and cytokine signaling [3]. About 40% of human TRIM proteins do not exhibit the PRY–SPRY domain, either individually or in combination [2]. Sardiello et al. proposed to divide the TRIM family into two groups that differed in domain structure and genomic organization: Group 1 members are present in both vertebrates and invertebrates and possess a variety of C-terminal domains, and Group 2 members display a C-terminal SPRY domain and are absent in invertebrates (Figure 1) [4]. Another classification based on domain organization has also been proposed, with TRIM proteins being classified in subfamilies ranging from I to XI (C-I to C-XI) [2].

TRIM proteins are implicated in many biological processes, including post-translational modifications, signal transduction, DNA repair, immunological signaling, autophagy, and oncogenesis, with the RING motif as an ubiquitin E3 ligase signature [2,5]. E3 ubiquitin ligases are key players in the physiology of the cell as well as for the pathology [6]. During ubiquitin-dependent protein degradation, a target protein is tagged with ubiquitins and subsequently degraded by the 26S proteasome. This process is instrumental for post-translational modifications and plays crucial functions in protein–protein interactions. Ubiquitination depends on an ubiquitin-activating enzyme (E1), an ubiquitin-conjugating enzyme (E2), and an ubiquitin ligase (E3) [5]. In this review, we focus on the role of TRIM37 in the normal cell and in pathological conditions, with an emphasis on the MULIBREY (MUscle-LIver-BRain-Eye).genetic disorder caused by *TRIM37* mutations.

## 2. TRIM37, Organization of the Gene, Gene Expression, Protein Structure

**Figure 2:**
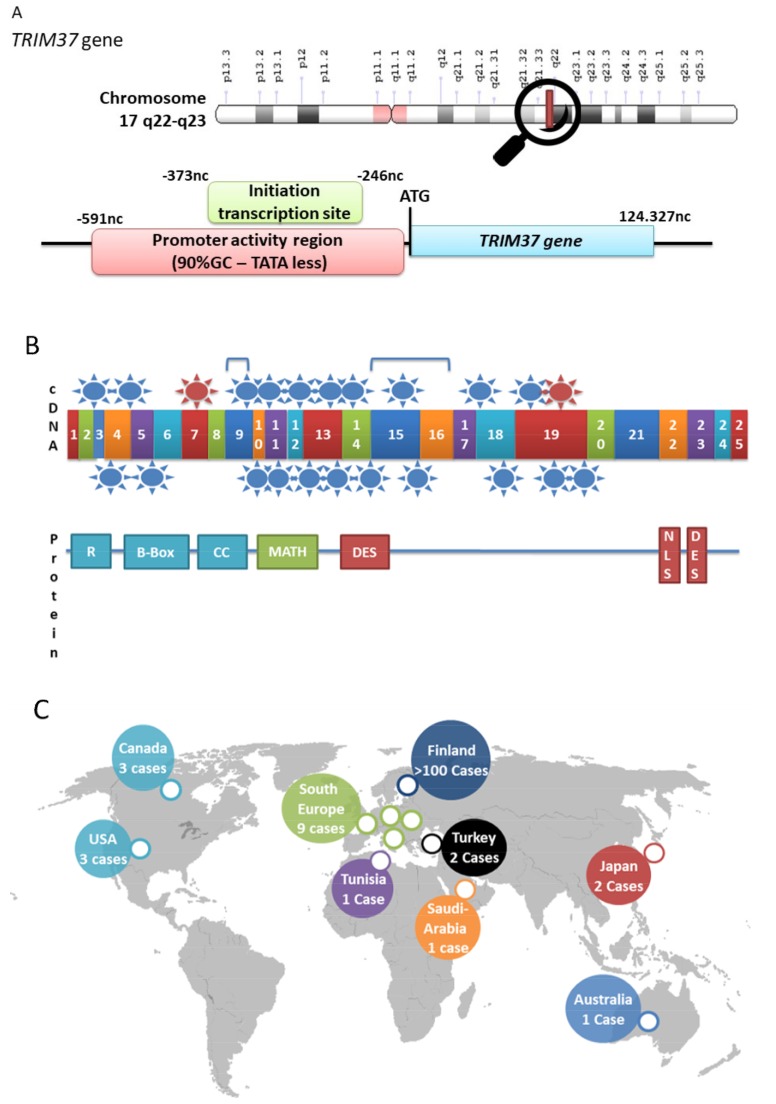
The *TRIM37* gene. (**A**) Schematic presentation of the MULIBREY-nanism-associated gene and *TRIM37* localization and gene structure. (**B**) cDNA, protein structure, and localization of mutations. Red circles correspond to the Finnish major and minor mutations; blue circles correspond to other mutations. Exons are numbered from 1 to 25. (**C**) Localization of mutations found in the *TRIM37* gene all around the world [8,9].

The human *TRIM37* gene is located in chromosome 17q22-23 and contains 25 exons, with one copy per haploid genome. The full genomic size is approximately 125 kb. The promoter region is G+C rich and TATA box less (Figure 2A). The major human transcript (called TRIM37a) encompasses 4.33 kb, encodes a 964 amino-acids protein, and is present in all tissues (Figure 2A,B) [7]. Amplification of the TRIM37 coding sequence by RT-PCR and in silico analysis indicates the presence of several alternative splicing products [7]. The main alternative transcript, TRIM37b, has the same coding sequence but differs in the 3’ UTR sequence. Its expression is much lower than the one of TRIM37a, except in testis where it is comparable [7]. Although there is a growing number of evidence that TRIM37 plays multiple physiological roles, *TRIM37* regulation has not been studied. The fact that mRNA levels of about half of the TRIM family members were found to be altered during viral infection suggests a tight control at both pre- and post-transcriptional levels. The presence of a RING finger domain suggests that TRIM37 gene/protein is a potential target for specific controllers in ubiquitin-dependent protein degradation [2].

MATH and DES domains are specific to TRIM37 in the TRIM family. The MATH domain is found in a small number of proteins and is only found in TRIM37 within the TRIM protein family (Figure 1). The MATH domain is specific to eukaryotes, including protozoa, suggesting an early appearance in evolution. The MATH domain is located on the C-terminal side of the coiled-coil region, encompassing 133 amino acids (273–406), and is formed by 7–8 antiparallel β-sheets [10]. It interacts with itself and can form hetero- and homo-oligomeric structures. The MATH domain is shared with other ubiquitin ligases, called TRAFs, and is involved in facilitating protein–protein interactions [11]. The MATH domain is thus also known as Tumor-necrosis-factor (TNF) receptor-associated factor (TRAF)-C domain. Proteins sharing the MATH/TRAF structure play an important role in protein processing, including proteolysis events and ubiquitination. This functional and structural identity suggests a common molecular link in the evolution within the TRAF and MATH family proteins. The primary structure of TRIM37 is represented in Figure 2B. TRIM37 displays two remarkable regions noted as DES-rich sequences (aspartate–glutamate–serine), localized between aa 423–555 and aa 873–943, respectively. These regions contribute to increased TRIM solubility, even among the hydrophilic amino acids [12]. Nuclear localization signals (NLS) is a short sequence motif that is related to protein transport out of and into the nucleus. 

## 3. MULIBREY Nanism is the Consequence of *TRIM37* Mutations

TRIM 37 is also known as the MULIBREY protein. MULIBREY nanism (muscle–liver–brain–eye) (MIM 253250) is a rare autosomal recessive disorder that affects several tissues of mesodermal origin, presenting with severe pre- and postnatal growth failure, characteristic dysmorphic features, but normal neurological development [8]. MULIBREY nanism is more common in Finland than elsewhere. The various mutations described in the literature are summarized in Table 1, with genomic localization, mutation type, and geographical origin. Mutations are summarized in Figure 2B. Figure 2C represents the geolocalization of patients all around the world. The so-called “Fin-major mutation” is a c. 493-2A > G transition in the 3’ splice site of exon 7 [13]. This mutation leads to a premature stop codon, resulting in a truncated protein of 174 amino acids. This mutation is present in nearly all the Finnish patients due to a founder effect. Another Finnish mutation has also been described (Fin-minor) that deletes one base pair (c.2212delG), giving rise to a premature stop codon and resulting in a truncated protein of 767 amino acids. Non-Finnish MULIBREY patients have been reported with other mutations, including new intragenic rearrangements and entire gene deletions [14,15]. We have described three composite heterozygous patients from North of France affected with the MULIBREY syndrome. They all shared a novel nonsense mutation (c.181 C > T p.Arg61*). Quantitative fluorescent multiplex PCR identified a new deletion of exons 15 and 16 in TRIM37 in one patient and another deletion of exon 9 in two siblings. Another non-Finnish composite heterozygous patient was also reported in Italy; Mutations were a 147-kb deletion inherited from the maternal allele and a mutation creating a new splice site in exon 19 that provoked a truncated protein and was inherited from the father [15]. Genetic rearrangements in *TRIM37* are likely due to the high number of Alu repeat sequences in introns (12). The phenotype of MULIBREY nanism is variable and overlaps with others, such as the Silver–Russell syndrome or the 3M syndrome. Up to now, 135 cases of MULIBREY nanism have been reported in the literature. 

## 4. MULIBREY Nanism and Cardiorenal Manifestations

Heart manifestations, including constrictive pericarditis, myocardial fibrosis, and myocardial hypertrophy, constitute the main common heart diseases of MULIBREY syndrome. Lipsanen-Nyman et al. studied a cohort of 49 male patients and followed them for up to 25 years. Half of them had congestive heart failure, more than a third had pericardiectomy for constrictive pericarditis, and 20% had died of cardiac causes. Of the patients, 19 were pericardiectomized and 12 among them benefited from lasting clinical benefit. Congestive heart failure recurred in approximately one third of patients. Autopsies showed a fibrotic thickening of pericardial leaves with myocardial hypertrophy and myocardial fibrosis [22]. Recently, a Japanese study confirmed the benefits of early diagnosis and total pericardiectomy before adherence of the pericardium [19]. Magnetic resonance imaging was used to assess structural and functional abnormalities of the MULIBREY cardiopathy in all four cardiac chambers as well as in the pericardium. The removed pericardium was found to be thickened and consisting of scar-like fibrosis. Left ventricular septum and posterior wall were found to be hypertrophied with a concomitant preservation of systolic function and a reduction of end-diastolic volumes of both ventricles [23]. A very severe case was described in Poland, where a28-year-old MULIBREY patient diagnosed with ovarian tumor could not undergo gynecological surgery because of pericardial constriction. She underwent pericardiectomy, and a calcified pericardium was found with deposits up to 8-mm thick in the heart. After cardiac surgery, the patient developed kidney insufficiency. Kidney complications are particularly common in MULIBREY patients [24]. Another study used levels of N-terminal proatriopeptide (ANPN) and N-terminal pro-brain natriuretic peptide (NT-proBNP) to estimate the overload of the left and right ventricle in children, including 25 MULIBREY patients. They found that in TRIM37-deficient children, NT-proBNP levels correlated negatively with left ventricle size [25].

**Figure 3:**
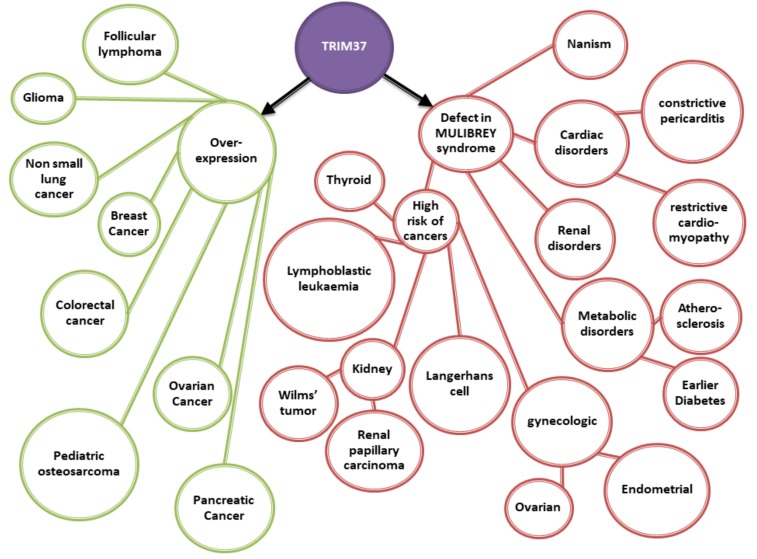
TRIM37 and pathology. MULIBREY patients, deficient in TRIM37, develop numerous pathologies, including various cancers. On the other side, TRIM37 overexpression is characteristic of various cancers found in the general population.

Metabolic alterations are also associated with the disease. When subjected to a 3-h oral glucose tolerance test, the MULIBREY patients had a regulation of glucose and lipid metabolism that varied wildly with age. The youngest patients had low fasting glucose and low insulin levels, while a vast majority of the adults patients (aged >20 years) had high fasting and postload insulin values. Among adult patients, 92% presented either with type 2 diabetes (50%) or with impaired glucose tolerance (42%). Metabolic syndrome was found in 70% of the adult patients [26]. There is a crosstalk between the insulin/insulin-like growth factor-1 (IGF-1) signaling and the ubiquitin pathways [27]. The insulin/IGF-1 is controlled by post-translational modifications, including ubiquitination, which declines during aging. Even if most MULIBREY patients have a normal kidney function, structural anomalies of the kidneys and urinary tract are found in 13% of the patients, concomitant with glomerular cysts, thick-walled blood vessels, and an increase in the expression of angiogenesis-related markers PDGF-B and FGF1 [28]. It is well known that long-term diabetes is associated with heart failure [19,29]. This seems to suggest a central role of TRIM37 in the development of metabolic disorders and, at the very least, of cardiorenal diseases (Figure 3).

A *TRIM37* knock-out mouse was described that recapitulates several features of the human disease. Mice were viable with normal weight until 12 months of age after which they experienced increasing weight loss, smaller skull size, with no difference in length of long bones. They presented with low fasting serum insulin level, elevated fasting blood glucose, enlarged and fatty liver, and cardiomyopathy.

Both sexes were infertile, with germ cell aplasia. Similar to MULIBREY patients, mice experienced a wide range of tumors [30]. 

## 5. TRIM37 Variants and the MULIBREY Syndrome, an Inflammatory Disease? The Role of TRIM37 in Innate Immunity and Antiviral Defense

Mutations leading to changes in the ubiquitination activity are related to genetic diseases, immune response, cellular transformation, and inflammatory responses. One of the main roles of post-translational modifications performed by ubiquitination is to select the fate of various proteins. Proteins are either targeted to proteasome for degradation or to participate in cell signaling pathways. Inflammatory pathways are tightly regulated by ubiquitination, and the human ubiquitin-mediated inflammatory signaling system ensures regulation of inflammatory responses [31]. Deregulation of ubiquitination has been shown to be instrumental in the progression of inflammatory diseases, such as obesity, insulin resistance, atherosclerosis, cardiac inflammation, and asthma. The association of TRIM37 with NF-κB signaling pathway has been demonstrated in non-small-cell lung cancer [32]. TRIM 37 induces K63 polyubiquitination of TRAF2, which is an important activator of NF-κB signaling. On the contrary, silencing TRIM37 leads to a decrease in mRNA expression levels of downstream genes in the NF-κB signaling pathway. These data strongly support the fact that TRIM37 plays an important role in the inflammation process by ubiquitination of NF-κB-signaling-related proteins. E3 ligases, including TRIM37, has been shown to be instrumental in the regulation of the inflammatory cascade [33]. The ubiquitin system (including TRIM family) has been studied in immune regulation. Indeed, protein ubiquitination is recognized as a crucial event in the control of the immune system, participating in the control of T-cell differentiation and signal transduction and leading to the induction of immune tolerance [34]. Furthermore, mutations in the autoimmune regulator gene (AIRE) that displays a E3 ligase activity lead to a breakdown in immune tolerance and development of autoimmunity [35]. Jian’s group cloned the coding sequence of all known 78 human TRIMs and showed that roughly half of the 78 TRIM family members, including TRIM37, were involved in innate immunity. TRIM family has been shown to enhance innate immune response levels in signaling pathways [36,37] The cellular localization was altered during viral infection, suggesting that the TRIM family of proteins has at least partly evolved as a component of innate immunity [2]. TRIM37 was the second member of the TRIM family (after TRIM5α) to be described as having anti-HIV-1 activity as its overexpression induced decreased viral replication and viral DNA synthesis [38]. TRIM37 was also shown to be involved in airway smooth muscle cell (ASMC) proliferation and migration, and thus in the progression of asthma [39]. TRIM37 expression was significantly decreased in proliferating airway smooth muscle cells, while overexpression of TRIM37 suppressed PDGF-BB-induced proliferation and migration by suppressing the Wnt/β-catenin signaling pathway, with repercussions in asthma.

The immune system must operate in an effective, precise, and safe manner to defend against diverse pathogens while avoiding attacking the body itself and commensal bacteria. Given the implication of TRIM37 in innate immunity described above, one could suspect that *TRIM37* mutations in MULIBREY patients could induce various immunity problems. 

A MULIBREY female patient with humoral immunodeficiency displayed subnormal concentration of serum IgG2 and IgG4 and low concentration of serum IgM. In contrast, IgA and IgD were elevated [40]. 

In young patients from North of France, we also found that IgM levels were decreased. Moreover, while the patients were vaccinated against tetanos, no vaccinal-specific Ig could be detected [14].

## 6. Infertility Problems in Both Sexes Are Found in MULIBREY Syndrome

MULIBREY patients are plagued with numerous infertility problems. Girls experience a transient minipuberty during childhood with a high follicle-stimulating hormone (FSH) surge that is normally found during mid-menstrual cycle in adult women where it will be responsible for ovulation. More than half never menstruate and only 8% of adult women menstruate regularly. In addition, ovaries are hypoplastic [41]. Concerning males, they all display small testes, elevated FSH and luteinising hormone (LH), and low inhibin B levels, indicating low spermatogenesis [42]. The same infertility problems and organ size are found in the murine model as both male and female *TRIM37* KO mice are infertile [30]. Infertility, in both male [43] and female [44], is partly due to inflammatory responses.

## 7. Nonsense-Mediated Decay is not Implicated in TRIM37 mRNA Expression in MULIBREY Syndrome

**Figure 4:**
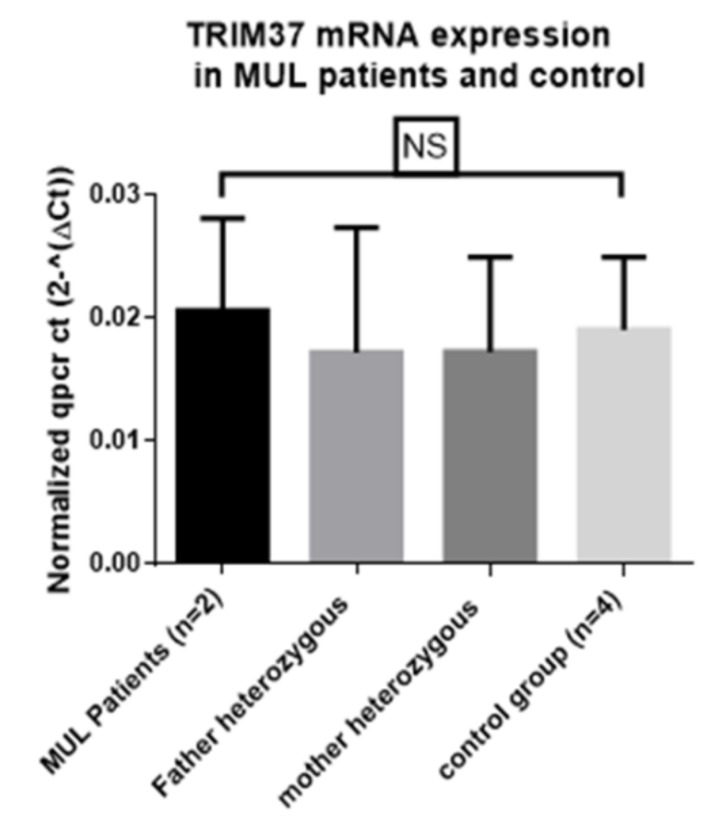
mRNA TRIM37 expression in MULIBREY patients (MUL), heterozygous parents, and control. NS: not significant. Total RNA was extracted in peripheral blood mononuclear cell by RNeasy minikit (QIAGEN). Retrotranscription were realized with cDNA transcription kit (Applied Biosystems) and qPCR was performed with Power SYBR green (Applied Biosystems) in Quanstudio7 (Thermofisher). TRIM37 primers sequence: AACAGAAGCGTGGAGAGCATT (exon1-2) and CTTCTGCCCAACGACAATTT (exon 4) described previously in Reference [14]. Results were normalized by 2^−∆Ct^ method with β-actin as reference gene as described previously [46]. This figure represents three independent experiments (Brigant et al., unpublished data).

RNA quality-control mechanism, named nonsense-mediated decay (NMD), recognizes and degrades mRNAs with premature translation termination codons [45]. NMD prevents the production of aberrant proteins. We quantified TRIM37 mRNA expression in MULIBREY patients with premature stop codons in two different alleles [14] and did not find any significant difference in TRIM 37 mRNA expression between MULIBREY patients, heterozygous subjects, and controls (Figure 4). In our MULIBREY patients, NMD system was unable to detect and degrade mRNA variants with a premature termination codon. 

## 8. TRIM37 Expression is Strongly Associated with Cell Proliferation and Cancer

*TRIM37* mutations are associated with a more frequent cancer diagnosis (Figure 4) as well as disturbed organ development [47], with a high number of gynecological tumors described in the literature [48]. For example, an Australian patient with mutations in both B-box and MATH domain altering the subcellular localization of TRIM37 was diagnosed with Wilms’ tumor [49]. The association of *TRIM37* mutations with increased cancer risk favors a prosurvival process in TRIM37-deficient tumor cells. Wang et al. found that lysosomal protein mTOR interacts with TRIM37, leading to transcriptional activation of genes involved in lysosome biogenesis and macroautophagy/autophagy. Autophagy may thus be a way for the cell to survive the loss of TRIM37 [50].

### 8.1. Cancerous Process is Associated with an Overexpression of TRIM37 in Various Tissues

Although *TRIM37* mutations lead to an increased risk of tumors [28], this is questionable when considering cancer among non-MULIBREY patients. In several non-MULIBREY cancers, *TRIM37* has been found to be overexpressed (Figure 3). 

TRIM37 plays a critical role in cell proliferation and, accordingly, its overexpression has been detected in a variety of human tumors (Table 2). Jian et al. described TRIM37 as being involved in the progression of both pancreatic and hepatic cancers [51,52]. TRIM37 expression was upregulated in hepatic cancer samples and was associated with advanced stage and tumor volume. TRIM37 overexpression was associated with poor outcomes and characterized as an independent prognostic factor for hepatic cancer. Authors found TRIM37 promotes hepatic cell migration and metastasis by activating the Wnt/β-catenin signaling. Similar results were found in pancreatic cancer. Colony formation assay and cell migration assay were performed to study the functions of TRIM37 in pancreatic cancer cells, and TRIM37 expression was increased in pancreatic tumor compared to normal tissue. TRIM37 overexpression promoted the migration and proliferation of pancreatic cancer cells in vitro by way of activation of the beta-catenin/TCF complex. The molecular mechanism study suggested that beta-catenin is the substrate of TRIM37 [51]. Both studies indicate an instrumental role of TRIM37 in cancer and its promising role as a target for hepatic and pancreatic cancer treatment. In colorectal cancer cell lines, overexpression of TRIM37 resulted in enhancement of proliferation, migration, and invasion via activation of the epithelial–mesenchymal transition pathway [53].

The genomic region hosting the *TRIM37* gene (17q23) is amplified in more than one third of breast cancers, and TRIM37 expression is increased in breast tumor. In contrast, the authors state that the major H2A ubiquitin ligase RNF2 (also known as RING1B) is downregulated. TRIM37 is associated with polycomb repressive complex 2, while RNF2 is associated with polycomb repressive complex 1 (PRC1), Both cases result in the silencing of specific target genes. The decrease in TRIM37 results in loss of ubiquitinated H2A and therefore a decrease in tumor growth, while overexpression of TRIM37 induces tumorigenesis [58].

Array comparative genomic hybridization in the 17q region identified highly frequent gain and loss of genetic material affecting more than 40% of ovarian cancers, suggesting that the MULIBREY gene is associated with ovarian tumorigenesis [61]. TRIM37 was also identified as directly related to the NF-κB pathway. As such, TRIM37 is linked to transformation of follicular lymphoma cells to a more aggressive disease, quick progression of the disease, and higher mortality [60]. A link between the NF-κB pathway and TRIM37 was also described in the context of non-small-cell lung cancer, where NF-κB is constitutively activated [32]. TRIM37 is a potential target for inducible chemotherapy resistance studies. It has been shown that TRIM37 activates NF-κB signaling via ubiquitination of NEMO (NF-κB essential modulator), which subsequently promotes cisplatin chemoresistance [62]. It should also be noted that TRIM37 knockdown inhibits proliferation and migration of glioma cells.

### 8.2. Accumulating In Vitro Evidences Point Toward Several Roles for TRIM37 in the Cell Cycle, Including in Peroxysomes and Centrioles

*TRIM37* was found to be one of the 44 genes indispensable for another part of cell division, the centriole formation [63]. Centrioles are microtubule-based particles that are templates for the formation of flagella and cilia, with roles in cell division, because it is microtubule-related. Centriole number is closely controlled as one new centriole is synthetized during each cell cycle [64]. Modifications in centriole biogenesis can alter centrosome number and thus affect genome integrity and mitosis, with repercussions for cell proliferation. A high-content, genome-wide small-interfering-RNA-based screen identified TRIM37 as instrumental in the prevention of centriole reduplication events and in the maintenance of genome stability. Depletion of TRIM37 resulted in chromosome segregation errors; TRIM37 prevents centriole reduplication [64]. The ubiquitin E3 ligase activity of TRIM37 was indispensable for this function. This is interesting in the light of MULIBREY disease as it has been documented that aberrations in centrosome number correspond with faster tumor progression in cancer patients [63]. 

TRIM37 localizes to peroxisomes and nucleus with an E3 ubiquitin ligase activity as a main function [65]. Peroxisomes are membrane-bound organelles in most eukaryotic cells, which react to physiological changes in their cellular environment and adopt their number, enzyme content, and metabolic functions accordingly. Wang et al. recently noted that TRIM37 ubiquitilates peroxysome proteins PEX5 and K464, which in turn promotes efficient peroxisomal targeting signal import [65]. It is interestingly to note that both TRIM37 and PEX5 deletion caused increased apoptosis. The authors gave the hypothesis that MULIBREY nanism is a new peroxisomal biogenesis disorder.

## 9. Concluding Remarks and Prospective

Dysregulation of TRIM proteins causes several types of diseases in humans. Inactivation of TRIM37 results in MULIBREY nanism and predisposes to both benign and malignant tumors, with several reports revealing a positive correlation between *TRIM37* overexpression and cancer. However, how patients with *TRIM37* mutations have a high risk of developing tumors still remains a challenging problem. Protein quality control and protein degradation are regulated by the ubiquitination proteasome, the NF-κB system, and autophagy. Further characterization of the signaling pathways that modulate TRIM37 expression and activity should help to understand the large repertoire of human pathologies in which TRIM37 is involved. Finding a way to keep equilibrium in the cellular expression of TRIM37 could lead to potential therapeutic treatments and future drug discoveries for developmental anomalies and cancer.

## Figures and Tables

**Table 1:** Summary of the mutations with genomic localization, geographical origin, and references.

n°	Mutations	Type	Exon	Country	Publish Cases	References
1	c.81delG	Deletion	2	French	1	[9]
2	c.181C > T	Nonsense	4	French	1	[14]
3	c.227T > C	Missence	4	Finnish	1	[16]
4	C.326G > C	Missence	5	Australian	1	[17]
5	c.493-2A > G	Splice-site	7	Finnish; Fin-major	>100	[13]
6	c.685_809del	Large deletion	9	French	1	[14]
7	c.745C > T	Nonsense	9	Canadian	1	[17]
8	c.810-1G > A	Splice-site	10	Turkish	3	[18]
9	c.838-842delACTTT	Deletion	10	Czech	1	[13]
10	c.860G > A	Splice-site	10	Australian	1	[17]
11	c.874_877delAGAG	Deletion	11	Japanese	1	[19]
12	c.965G > T	Missence	12	Canadian	1	[17]
13	c.1016C > G	Missence	13	Japanese	1	[19]
14	c.1037_1040dupAGAT	Duplication	13	Canadian	1	[17]
15	c.1166A > G	Nonsense	13	Finnish	1	
16	c.1233delA	Deletion	14	German	1	[20]
17	c.1313+507_1668-207del	Large deletion	15,16	Italian	1	[17]
18	c.1315_1667del	Large deletion	15-16	French	1	[14]
19	c.1346dupA	Duplication	15	American	1	[13]
20	c.1411C > T	Nonsense	15	Canadian, Tunisian	1	[17]
21	c.1894_1895delGA	Deletion	18	Turkish	1	[21]
22	c.1910_1911dupTA	Duplication	18	Swiss	1	[9]
23	c.1949-12A > G	Splice-site	19	Italian	2	[15]
24	c.2056C > T	Nonsense	19	Saudi-Arabian	1	[17]
25	c.2212delG	Deletion	19	Finnish; Fin-minor	1	[13]
26	c.1949-12A > G	Splice-site	19	Italian	2	[15]

**Table 2:** TRIM37 dysregulations in Cancer.

Cancers	Publications	Signaling Pathways	Known Substrates	Association with:	TRIM37 Expression in Tumor Cells
Pediatric osteosarcoma	[54]	Wnt/β-catenin	-	Proliferation and drug resistance	Overexpressed
Glioma	[55]	PI3K/Akt	-	Proliferation, migration, and invasion	Overexpressed
Non-small-cell lung cancer	[32]	NFκB	Ubiquitinates TRAF2	Apoptosis, angiogenesis, and proliferation	Overexpressed
Pancreatic cancer	[51]	Wnt/β-catenin	Binds β-Catenin	Proliferation and migration	Overexpressed
Hepatocellular carcinoma	[52]	Wnt/β-catenin	-	Proliferation and migration	Overexpressed
Colorectal Cancer	[53]	-		Proliferation, invasion, and migration	Overexpressed
	[56]	Wnt/β-catenin	-	Proliferation	Overexpressed
Breast Cancer	[57,58]	PRC1/PRC2	H2A ubiquitination	Promotes transformation/silencing of tumor suppressor	Overexpressed in ∼40% of breast cancers
	[59]	-	-	High frequency of chromosomal rearrangement in *TRIM37* gene associated with mortality	-
Follicular lymphoma	[60]	NFκB	-	Promotes transformation	Overexpressed
Ovarian Cancer	[61]	-	-	High frequency of chromosomal rearrangement in *TRIM37* gene	-
Esophagus Cancer	[62]	NFkB	Binds TRAF6 and ubiquitinates NEMO	Relapse-free survival and drug resistance	Overexpressed
